# Overexpression of the *Rybp* Gene Inhibits Differentiation of Bovine Myoblasts into Myotubes

**DOI:** 10.3390/ijms19072082

**Published:** 2018-07-18

**Authors:** Xiaotong Su, Yanfang Zhao, Yaning Wang, Le Zhang, Linsen Zan, Hongbao Wang

**Affiliations:** 1 College of Animal Science and Technology, Northwest A&F University, 22th Xinong Road, Yangling 712100, China; xiaotongsu86@gmail.com (X.S.); zhaoyanfang@novogene.com (Y.Z.); wangyn1992@gmail.com (Y.W.); zhangle@nwsuaf.edu.cn (L.Z.); zanls@nwsuaf.edu.cn (L.Z.); 2 National Beef Cattle Improvement Centre, Yangling 712100, China

**Keywords:** cattle, *Rybp* gene, myoblasts, myotubes, adenovirus, RNA-seq

## Abstract

RING1 and YY1 binding protein (*Rybp*) genes inhibit myogenesis in mice, but there are no reports on the effects of these genes in cattle. The aim of this study is to investigate the roles of the *Rybp* gene on bovine skeletal muscle development and myoblast differentiation. In the present study, the *Rybp* gene was overexpressed in bovine myoblasts via adenovirus. RNA-seq was performed to screen differentially expressed genes (DEGs). The results showed that overexpressing the *Rybp* gene inhibits the formation of myotubes. The morphological differences in myoblasts began on the second day and were very significant 6 days after adenovirus induction. A total of 1311 (707 upregulated and 604 downregulated) DEGs were screened using RNA-seq between myoblasts with added negative control adenoviruses (AD-NC) and *Rybp* adenoviruses (AD-Rybp) after 6 days of induction. Gene ontology (GO) and KEGG analysis revealed that the downregulated DEGs were mainly involved in biological functions related to muscle, and, of the 32 pathways, those associated with muscle development were significantly enriched for the identified DEGs. This study can not only provide a theoretical basis for the regulation of skeletal muscle development in cattle by exploring the roles of the *Rybp* gene in myoblast differentiation, but it can also lay a theoretical foundation for molecular breeding of beef cattle.

## 1. Introduction

With economic development, peoples’ demand on the nutritional value of food is increasing. Beef is rich in protein, iron, selenium, zinc, copper, and manganese, and it also has a lower fat content than pork, meaning that beef can better meet human needs for amino acids. Beef not only improves the body’s immune function but is also suitable for people who want to supplement blood loss and repair tissue. These reasons have led to beef being increasingly favored by Chinese consumers [1,2,3,4,5,6]. As demand for beef grows in China, beef prices continue to rise. Developing methods to improve the yield and quality of beef has become the main focus of research in cattle breeding. Studies of the mechanisms regulating the development of beef muscle tissue will reveal their effects on meat production performance and beef quality at the molecular level and lay the foundation for molecular breeding of beef cattle. The formation of muscle is regulated by a series of proteins, including WNT1, PAX3, PAX7, and myogenic regulatory factors (MRFs) [7,8]. PAX7 activates the muscle satellite cells [9]. PAX3 and PAX7 affect the expression of MRF4, MYOD, and MYF5 in myoblasts. MYOD and MYF5 activate specific genes in muscle that regulate the differentiation of progenitor cells into muscle cells and the formation of myotubes [10,11,12,13,14]. In addition to these transcription factors, epigenetic regulation also plays an important role in transcriptional regulation of myogenesis, and some important signaling pathways in loci related to muscle are formed by the interaction of transcriptional and epigenetic regulation [15].

RING1 and YY1 binding protein (Rybp), also known as death effector domain-associated factor (DEDAF), is an alkaline protein with a zinc finger and domain composed of 228 amino acid residues [16]. Based on biophysical and hydrodynamic techniques, Rybp is a natively unfolded protein. When it interacts with protein or DNA, Rybp acquires a folded conformation [17]. Rybp can interact with proteins such as RING1, YY1, E2F2, and E2F3 and can regulate transcription. Rybp is distributed in the cytoplasm and nucleus and has the highest expression in human lymph tissue and placenta [18]. It has many biological functions and can interact with many proteins in the cytoplasm and nucleus to regulate apoptosis [19]. Studies on model animals, including flies and mice, have shown that Rybp plays a role in the development of the embryonic, ocular, and central nervous systems [20]. Many studies have shown that Rybp is closely related to the development of human malignant tumors [19]. As a member of the comb gene family, Rybp is involved in the composition of polycomb repressive complex 1, and plays an important role in inhibiting gene transcription, regulating embryonic development, and epigenetic inheritance [21].

The formation of muscle is a complex biological process. Studies have shown that microRNA (miRNA) also plays a role in the regulation of myogenesis by inducing mRNA division and inhibiting translation by binding to its target 3′ untransliated region(UTR), negatively controlling its target genes at post-transcriptional levels. Among miRNAs, miRNA-29 is a myogenic-derived factor that interacts with YY1 and EzH2 [22]. Studies have shown that YY1 is activated by nuclear factor kappa-light-chain-enhancer of activated B cells (NF-κB) and assocoiates with EzH2 to silence miRNA-29 transcription, inhibiting muscle differentiation [23,24]. In the miRNA-29 genome transcriptional regulatory network, the miRNA-29 gene can regulate the transcription level of the *Rybp* gene by binding with the 3′-UTR of the *Rybp* gene, making Rybp one of the targets of miRNA-29. Functional studies showed that Rybp expression was downregulated during the differentiation of C2C12 cells and muscle regeneration in vivo, which led to negative regulation of the formation of skeletal muscle [24]. In addition, Rybp and YY1 share some of the sites of myogenesis, including the miRNA-29 gene; this silences the miRNA-29 gene, thus forming the Rybp-miR-29 feedback loop. Overexpression of Rybp increases EzH2 content and H3K27 methylation at the target site, suggesting that the *Rybp* gene may promote the complementation and stabilization of the comb-inhibiting complex. These results indicate that the *Rybp* gene can silence the miRNA-29 gene and other myogenic loci by co-acting with YY1, thus playing a regulatory role in myogenesis [25].

Although the relationship between the *Rybp* gene and skeletal muscle production has been verified in mice, research on the *Rybp* gene in livestock animals is minimal, and its function is still unclear. In our previous studies, the expression level of the bovine *Rybp* gene was much higher in testis than in other tissues, whereas the expression level in longissimus dorsi muscle (LDM), tenderloin, and heart was relatively low [26]. During the growth and development of Qinchuan cattle, the expression level of the *Rybp* gene in muscle tissue increased gradually, while it significantly decreased in adipose tissue. At different stages of muscle cell differentiation, the mRNA expression level of the *Rybp* gene gradually increased as differentiation of myoblasts accelerated, while the expression level of *Rybp* decreased when the differentiation slowed down. In the present study, adenovirus-mediated overexpression was used to detect the function of Rybp in regulating differentiation of bovine myoblasts. In addition, in order to further explore the molecular mechanism of Rybp on bovine myoblast differentiation, RNA-seq was performed to screen for differentially expressed genes (DEGs). The aim of this study is to investigate the roles of the *Rybp* gene on bovine skeletal muscle development and myoblast differentiation. This study can provide both a theoretical basis for the regulation of skeletal muscle growth and development and a foundation for molecular breeding of beef cattle.

## 2. Results

### 2.1. Determination of Recombinant Adenovirus Multiplicity of Infection (MOI) Value

**Figure 1:**
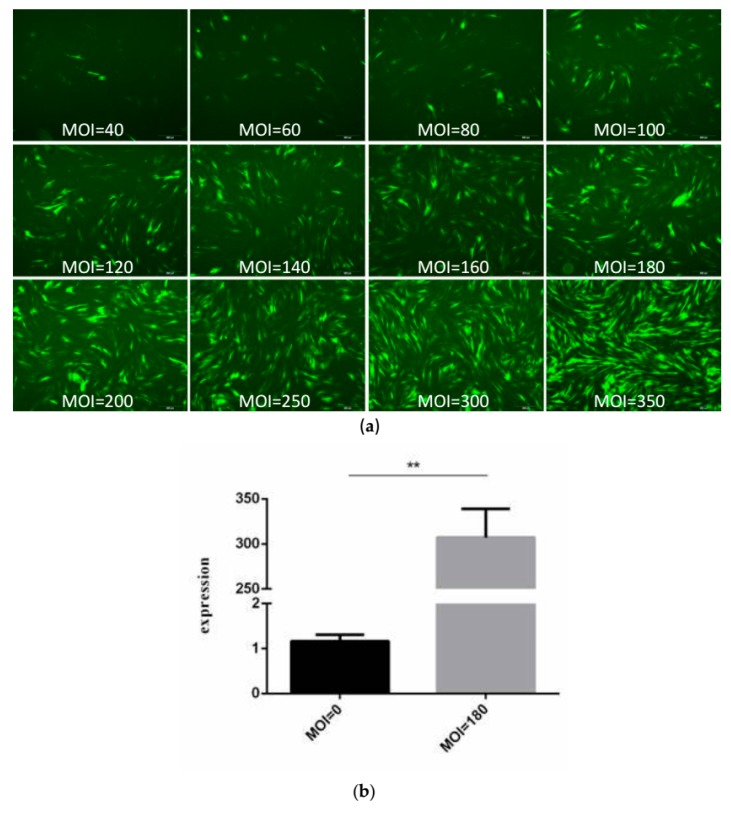
Determination of recombinant adenovirus multiplicity of infection (MOI) value. (**a**) Different MOI values 72 h after *Rybp* adenovirus (pAd-Rybp) infection of bovine myoblast (40× magnification); (**b**) the expression of the *Rybp* gene in myoblasts with 0 and 180 MOI of pAd-Rybp. (** *p* < 0.01).

The optimal multiplicity of infection (MOI) value is the quantity of transfected virus with the highest fluorescence intensity, the same cell morphology, and the highest expression level of the *Rybp* gene. As the MOI value increases, fluorescence is increasingly enhanced; when the MOI of the recombinant adenovirus was 180, the fluorescence intensity was strong, meaning the myoblasts grew well (Figure 1a). When AD-Rybp with an MOI value of 180 was added to the myoblasts, the expression level of the *Rybp* gene was about 300 times higher than that of the non-enriched virus (Figure 1b). The difference was extremely significant (*p* < 0.01). In brief, when the MOI value is 180, the virus can be used for subsequent experiments [27]. 

### 2.2. Effects of Overexpression of the Rybp Gene in Myoblasts

**Figure 2:**
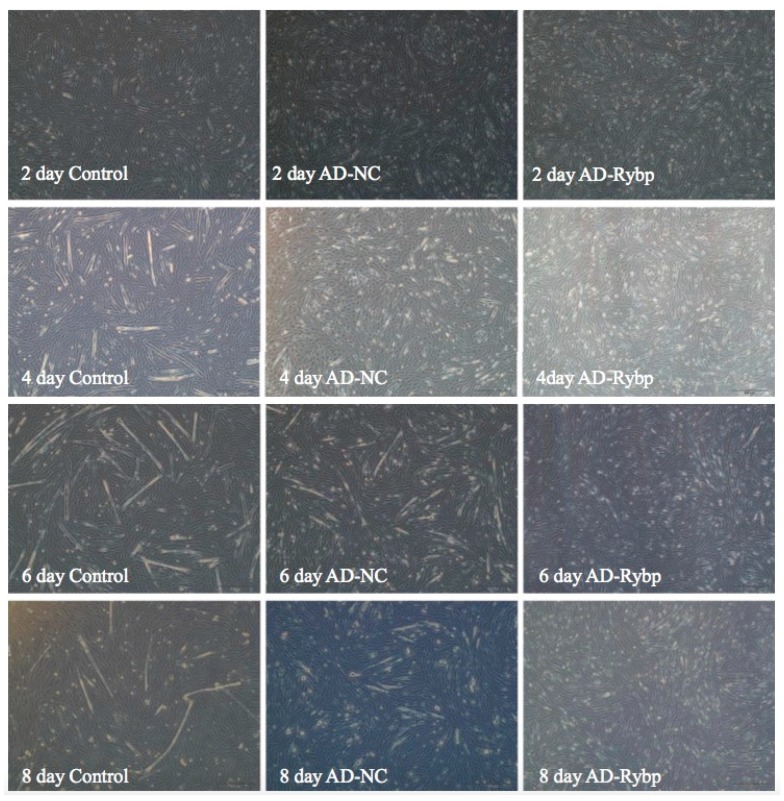
The morphologic changes of myoblasts at different differentiation times (2 days, 4 days, 6 days, and 8 days) after overexpressing the *Rybp* gene (40×). Note: the differences in myoblast fusion were visible after 4 days of treatment; the AD-Rybp cells were poorly fused (fewer and shorter myotubes) compared to cells with added negative control adenoviruses (AD-NC) or the control group.

To determine whether overexpression of Rybp could influence differentiation of myoblasts into myotubes, changes in myoblast morphology were investigated over time after Rybp was overexpressed and differentiation of myoblasts was induced. Results of the myoblasts cultured with added negative control adenoviruses (AD-NC) were compared with those of the control group. Four days after treated, the AD-Rybp myoblast group showed less-fused myotubes compare to the other two groups (AD-NC and control group), and such phenotypic changes become more visible 6 days after treatment. A much greater fusion of myoblasts and longer myotubes were observed in the two control groups, while in the myoblasts treated with AD-Rybp, few myoblasts were fused into myotubes, and only a few short myotubes could be seen (Figure 2). The differentiation of myoblasts treated with AD-Rybp was significantly less than myoblasts treated with AD-NC or the control group; the most significant difference occurred on day 6.

### 2.3. Deep Sequencing Analysis of the Differentiation Inhibition Process

**Figure 3:**
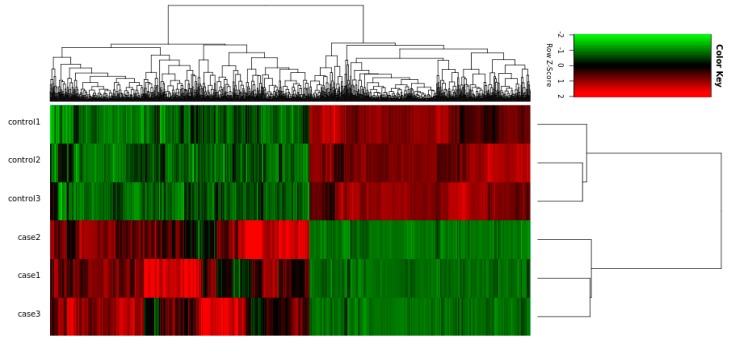
Heatmap for the hierarchical cluster analysis of differentially expressed genes (DEGs) between samples. Control1–3 is to add the recombinant adenovirus AD-NC sample; Case1–3 is to add the recombinant adenovirus AD-Rybp sample. Red represents upregulated genes and green represents downregulated genes.

**Figure 4:**
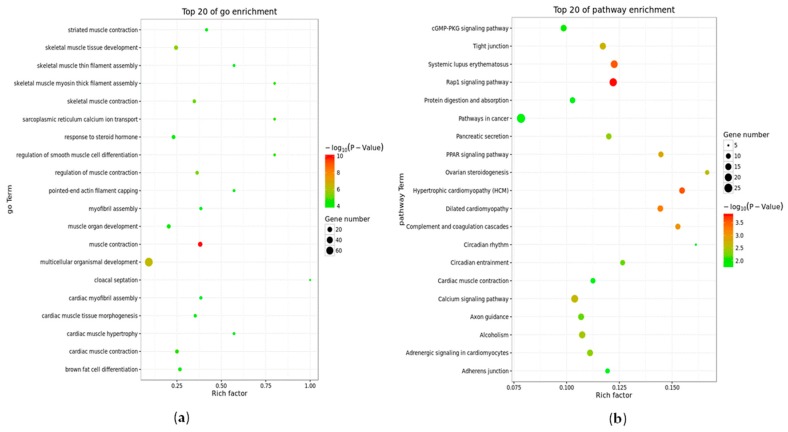

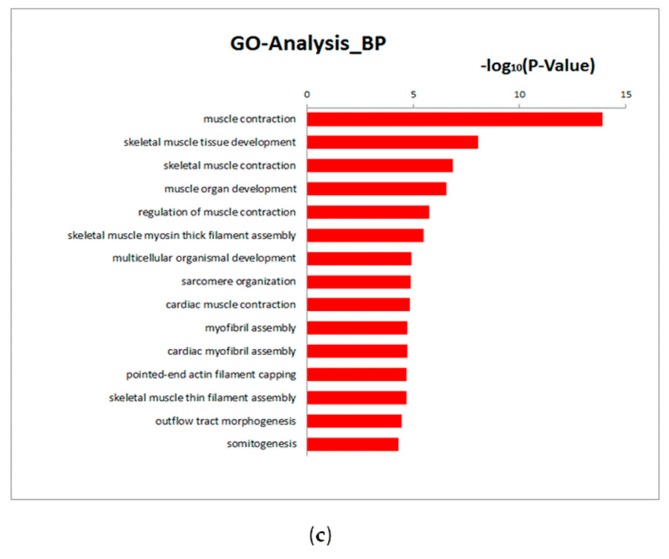
Gene ontology (GO) and pathway analysis of DEGs. (**a**) Top 20 significant GO terms (biological processes) associated with the identified DEGs. The vertical axis represents the GO category, and the horizontal axis represents the −log_2_ (*p*-value) of the significant GO terms; (**b**) Top 20 significant pathways involving DEGs. The vertical axis represents the pathway category, and the horizontal axis represents the −log_2_ (*p*-value) of the significant pathways. Greater −log_2_ (*p*-value) scores are correlated with increased statistical significance; (**c**) Top 15 significant GO terms (biological processes) associated with the identified downregulated DEGs.

In order to reveal the mechanisms of how Rybp inhibits differentiation of myoblasts into myotubes, RNA sequencing was performed to profile the genes expressed during the myoblast differentiation process. We examined the gene expression profiles on the sixth day of induced differentiation. In total, 1311 genes were identified as differentially expressed between the AD-Rybp group and the AD-NC group after 6 days of infection. Among these genes, 707 genes were significantly upregulated, and 604 genes were significantly downregulated (Figure 3). The DEGs were classified into categories by cellular component, molecular function and biological process using gene ontology (GO) annotation. Most of the genes were correlated to multicellular organism development, and the most significant GO term was muscle contraction (Figure 4a). The downregulated genes were mainly correlated to muscle contraction, skeletal muscle tissue development, skeletal muscle contraction, muscle organ development, regulation of muscle contraction, skeletal muscle myosin thick filament assembly, multicellular organism development, and so on (Figure 4c). According to the KEGG analysis, most of the DEGs are involved in pathways related to cancer, calcium signaling, peroxisome proliferator-activated receptor (PPAR) signaling, Rap1 signaling, systemic lupus erythematosus, and so on (Figure 4b). However, many downregulated genes were still correlated to the PPAR signaling pathway, calcium signaling pathway, and Wnt signaling pathway, with 76, 183 and 150 DEGs, respectively.

### 2.4. Identification and Validation of the Differentially Expressed Genes during Inhibition of Differentiation Based on qRT-PCR

**Figure 5:**
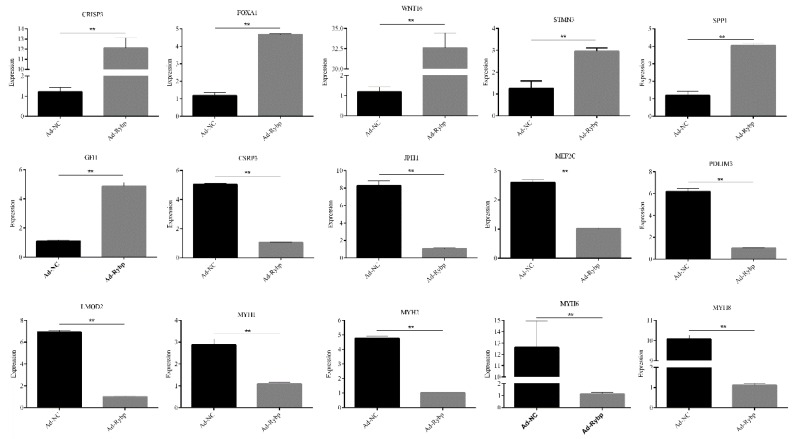
qRT-PCR validation of relative expression levels of representative DEGs. The measured RNA expression levels were normalized by GAPDH. Average values of three replicates are given with the mean ± SD (*n* = 3) (** *p* < 0.01).

To confirm the results of RNA-seq, we used qRT-PCR to validate changes in expression levels of myoblasts treated with AD-Rybp with induced differentiation. Glyceraldehyde 3-phosphate dehydrogenase (GAPDH) was used to normalize the gene expression (Figure 5), and all DEGs were confirmed by real-time PCR. All DEGs had significant differences (*p* < 0.01) between AD-Rybp and AD-NC. 

## 3. Discussion

In recent years, the regulation of muscle cell differentiation, growth, and development has been explored at the molecular level. A previous study by Zhou et al. [25] showed that Rybp inhibits myogenesis by acting with miRNA-29 in mice, but the role of Rybp in myoblast differentiation has not been tested in livestock animals. In this experiment, we overexpressed the *Rybp* gene in myoblasts to verify whether it plays a key role in myoblast differentiation in Qinchuan cattle.

AD-Rybp and AD-NC were infected into myoblasts, which induced differentiation, and the morphological differences between the experimental group and control group were observed under a microscope. The results showed that myoblasts with AD-NC and control myoblasts gradually differentiated into myotubes as differentiation time increased, ultimately differentiating to myotubes in 6 days at most, after which there was no further increase. Results also showed that the difference between control myoblasts and those with AD-NC was not obvious. With AD-Rybp, there were almost no myoblasts differentiating into myotubes, which was significantly different from the control group. The experimental and control groups only differed in recombinant adenovirus, and thus, overexpressing the *Rybp* gene in myoblasts led to weak differentiation ability, even to the point where differentiation does not occur. Thus, the *Rybp* gene may have an inhibitory effect on myoblasts’ ability to differentiate into myotubes. The findings from the present study show that Rybp expression was downregulated during the differentiation of C2C12 cells, further confirming our previous results [25].

In order to further explore the molecular mechanism of *Rybp* gene inhibition on bovine muscle cell differentiation, this experiment focused on screening significant DEGs between the experimental and control group by RNA-seq, and the RNA-seq results were verified using qRT-PCR.

GO analysis showed that most DEG functions were related to muscle function*,* particularly in muscle contraction, suggesting that the *Rybp* gene is related to the development and assembly of muscle tissue. Another interesting result is, compared to upregulated DEGs, many more downregulated DEG genes (including *MYOG*, *MYF6*, and *MEF2C*) were enriched in biological function, including muscle contraction and skeletal muscle tissue development, which suggests that *Rybp* may play its role upstream of the muscle development signal pathway. Its downstream genes were reported to be crucial for muscle growth and muscle cell differentiation. Mice lacking the *MYOG* gene still have a muscle system through myoblast formation, however, these mice are susceptible to death before or after birth because of severe impairment to myoblast differentiation and muscle fiber formation, indicating that MYOG is a differentiation factor during myogenesis [28,29,30]. In vertebrates, myogenic precursor cells originating from somites proliferate and differentiate into myoblasts, and then myoblasts differentiate into multinucleated myotubes after a series of activities [31,32]. This process is precisely regulated by the synergistic action of MYOG, MYOD, MYF5, and MYF6, which belong to the MRF family of transcription factors regulating muscle-related gene expression [33]. Although MRF family members have overlapping expression patterns, each member plays a different role in myoblast differentiation [34]. MYOG and MYF6 regulate adult terminal differentiation and myotube formation [35,36]. MEF2C is a necessary factor for cardiac myogenesis and right ventricular development [37]. The above results indicate that the expression of genes related to muscle tissue development and assembly are downregulated after overexpressing the *Rybp* gene in myoblasts, which suggests that the *Rybp* gene can inhibit the expression of genes related to assembly and muscle development to inhibit myoblasts differentiating into myotubes and muscle development.

According to the KEGG analysis, the top 20 significant pathways involving DEGs include the PPAR signaling pathway and hypertrophic cardiomyopathy, dilated cardiomyopathy, and the calcium signaling pathway, all of which are related to muscle development. Some downregulated DEGs were enriched in the PPAR signaling pathway, the calcium signaling pathway, and the Wnt signaling pathway. In the pathway network, the relationship between signaling pathways is complex. The Wnt pathway, adherens junction pathway, and Ca signaling pathway have strong relationships with muscle tissue development, especially the Wnt signaling pathway. Muscle development and myogenesis are regulated by a series of signaling pathways, and normal muscle differentiation involves the expression of MRF family members, such as MYF5, MYOD, MYOG, and PAX3/7. Research shows that in the stages of prenatal and postnatal muscle development, the Wnt ligand regulates the expression of MRF family members to activate the signaling pathway that regulates muscle growth and myogenesis and the formation of multinucleated myotubes [38]. While the Wnt signaling pathway was in a reduced state in the signal pathway network, we speculate that the overexpression of the *Rybp* gene can downregulate the Wnt signaling pathway to inhibit the expression of MRF family members, so that differentiation of myoblasts into myotubes is inhibited. Some functional genes can increase the body measurement traits (BMTs) of beef cattle and accelerate breeding efforts related to these traits for Qinchuan cattle breed improvement [39]. For example, Wei et al. [40] found that the gene *SIX1* not only mediates skeletal muscle growth by regulating the myogenic regulatory factors MYOD and myogenin, but also improves meat quality. In our study, the *Rybp* gene was shown to be closely related to muscle development, and we speculate that the inhibition of muscle growth by the *Rybp* gene can be applied for achieving the goal of accelerating the growth rate and improving the yield and quality of beef cattle through genetic engineering techniques.

In conclusion, we report that Rybp is a negative regulator of skeletal muscle myoblast differentiation and speculate that Rybp regulates myoblast differentiation via inhibiting the expression of genes such as melanin, MYF6, MEF2C, and Wnt signaling pathways.

## 4. Materials and Methods

### 4.1. Isolation and Culture of Bovine Myoblasts

Isolation and cell culture of bovine myoblasts were performed as described by Wang et al. [27]. Simply, the myoblasts were isolated from the hind limb muscle and cultured in growth medium (containing DMEM/F-12, 20% FBS, 1% penicillin/streptomycin). When the bovine myoblasts reached 70% confluency, the growth medium was switched to differentiation medium (containing DMEM/F-12, 2% horse serum, 1% penicillin/streptomycin). The cell culture medium was changed every 2 days.

### 4.2. Vector Construction and Recombinant Adenovirus Packaging

The adenovirus AdEasy system is a simple and convenient adenovirus recombination system constructed by He in 1998, and has been widely used in scientific research [41]. The system is composed of an adenovirus shuttle vector (pAdTrack or pShuttle) and a backbone vector (pAdEasy). First, the target gene was inserted into a shuttle vector (such as pAdTrack-CMV), followed by a recombinant adenovirus created by homologous recombination in bacteria with a skeleton vector, linearization of adenovirus, and transformation.

Adenovirus vectors were constructed as previously described [41]. Fragments containing the complete coding sequence (CDS) of the bovine *Rybp* gene or the empty pAdTrack vector (Takara Biomedical Technology (Beijing) Co., Ltd, Dalian, China) were ligated with AdEasy expression vector (Takara Biomedical Technology (Beijing) Co., Ltd, Dalian, China), and the subsequent adenoviruses—AD-Rybp and AD-NC—were produced in the HEK 293A cell line [42].

### 4.3. Detection of Optimal MOI Value

Before testing, the myoblasts were cultured in 6-well plates. The MOI values were 40, 60, 80, 100, 120, 140, 160, 180, 200, 250, 300 and 350. One-hundred microliters, 150 μL, 200 μL, 250 μL, 300 μL, 350 μL, 400 μL, 450 μL, 500 μL, 550 μL, 600 μL, or 650 μL of recombinant adenoviruses were added accordingly. Three biological replicates were conducted for each group. After 3 days of cell culture, the optimal MOI value was selected based on changes in cell morphology and real-time fluorescence quantitative detection of the expression level of the *Rybp* gene.

### 4.4. Overexpression of Rybp in Bovine Myoblasts

When the bovine myoblast confluence reached approximately 80%~90%, they were infected with AD-Rybp and AD-NC recombinant adenovirus, respectively. Normal culture cells without any treatment were selected as control group. The morphologic changes of myoblasts at different differentiation times (2 days, 4 days, 6 days, and 8 days) were observed by light microscopy. 

### 4.5. RNA-seq

After 6 days of treatment, the bovine myoblasts were harvested, RNAs from three biological replicates of each group were isolated using RNeasy kit (Qiagen China (Shanghai) Co Ltd, Shanghai, China), and the quality was analyzed using an Agilent 2100 bioanalyzer (Agilent technologies, California, CA, USA). The gene expression profiles of AD-Rybp and AD-NC groups were investigated using Illumina HiSeq2000 RNA Sequencing according to the manufacturer’s guide (Illumina, San Diego, CA, USA).

### 4.6. Identification of DEGs

DEGs were screened using an algorithm according to Wang et al. [43], and the Benjamini–Hochberg (BH) procedure [44] was performed for multiple testing correction. Genes with an absolute fold change greater than 2.0 and a false discovery rate (FDR) less than 0.05 (FDR ≤ 0.05) were selected to be differently expressed [45]. The heatmap was constructed using Cluster 3.0 (http://bonsai.hgc.jp/~mdehoon/software/cluster/software.htm), which can perform hierarchical clustering analysis, and the results were visualized using the Java TreeView program [46]. 

### 4.7. Functional Enrichment Analysis

To understand the gene functions among the selected DEGs, GO functional enrichment was carried out based on the NCBI Gene Ontology database [47] using the methods described by Wang et al. [48]. Pathway enrichment analysis was conducted according to the KEGG database (http://www.genome.jp/kegg) and using KegArray software [49]. Fisher’s exact test was applied to identify significant GO categories and enriched pathways. For the GO categories, *p*-values of less than 0.01 after correction by FDR were selected. For the KEGG pathway terms, corrected *p*-values of less than 0.05 were screened out. In addition, Pathway-Act-Networks were constructed on the basis of the relationships between the pathways in the KEGG database.

### 4.8. Quantification of mRNA Expression by Using RT-PCR

Total RNA of the bovine myoblasts was extracted by using the Total RNA Kit I (OMEGA Bio-Tek, Guangzhou, China) according to the manufacturer’s instructions. After quality detection by agarose gel electrophoresis assay and spectrophotometer assay, the total RNA was applied for reverse transcription reactions to synthesize cDNA by using Prime Script RT reagent Kit with gDNA Eraser (TaKaRa, Dalian, China). A quantitative real time-PCR (qRT-PCR) experiment was then performed three times (in triplicate wells) by using the SYBR^®^ Premix Ex Taq^TM^ II kit (TaKaRa, Dalian, China) in ABI 7500 Real Time PCR System (ThermoFisher Scientific, Waltham, MA, USA). After selection of the optimal endogenous control gene with NormFinder, GeNorm (version 3.4), and Bestkeeper software (version 1), GAPDH was finally chosen for normalizing the relative mRNA expression level [50]. The experiment data were analyzed by using the 2^−ΔΔ*C*t^ method. Summary information of the primers used for qRT-PCR detection are listed in Table 1.

## Figures and Tables

**Table 1:** Sequence information of the primers used for real-time PCR.

Gene	Forward Primer (5′-3′)	Reversed Primer (5′-3′)	Fragments Size
*WNT16*	AGGAAACTGGATGTGGTTGG	GGTTTCCTCTTGCACAGCTC	108
*FOXA1*	AAGATGGAAGGGCACGAGAG	CCAGACCCGAGTTCATGTTG	100
*GFI1*	CCAGAGCTCCAACCTCATCA	GAGGTCCACCTTCCTCTGGA	100
*DPPA2*	GGAACTGGTGCCAACAACTT	CCTCCCGTGATGATTTAGGA	120
*CRISP3*	GGATCGACGTTTTGCTGATT	TGTTCCATTGCATCTTCAGC	128
*STMN3*	GCTCATCTGCTCCTGCTTCT	AGGGGACTTGAGGATGACCT	127
*ELAVL3*	CAAACTGCAGACGAAGACCA	CCGTACTGGGAGAAGAGCTG	138
*MANEAL*	CCTCCCATCAGAACTGGAAA	ACTTGCCATTGACCCTGTTC	144
*MYH1*	GACAACTCCTCTCGCTTTGG	GCCTTCAGCTGGAAAGTGAC	122
*MYH2*	CTGGCTGGAGAAGAACAAGG	TGCCTCTGAGTCACCAGTTG	118
*MYH4*	ATTGACATCCTGGGCTTCAC	TCAGCAACTTCAGTGCCATC	140
*MYH8*	GGAAAGATCCTGCGTATCCA	GGTTCCTCTTCAGCTGTTCG	103
*FGFR2*	AGGTACGAAACCAGCATTGG	GATTGATGGACCCGTAATCG	102
*MEF2C*	TCCTGATGCAGACGATTCAG	TCAAAGTTGGGAGGTGGAAC	126
*LMOD2*	TCGAGTCCAACTTCATCACG	AGCAGCTTGACGATCTCCAT	142
*CSRP3*	CAGTGCAATGGGAGGAGTTT	GCCCGTAGCAGACCTTACAG	121
*JPH1*	GCCTCACTTCTACCGCAAAG	AACTGGGGTTCTGCTTCCTT	107
*PDLIM3*	ACAACCGCAATGAACCTACC	GCTCTCACACTCCGAGTTCC	112
*MYF6*	AATGTCTGATCTGGGCTTGC	GGCCTCGTTGATTTTCTTGA	110
*TNFRSF9*	TGAGAGGACACGATCACTGC	CTGTCCACTTGTGCTGGAGA	121
*BMP4*	GGATCAGGGCTTTCATCGTA	TCACATTGTGGTGGACCAGT	116
*SPP1*	CAGAGTCCAGATGCCACAGA	GGAAAGCTCGCTACTGTTGG	126
*GAPDH*	ACACCCTCAAGATTGTCAGC	GTCATAAGTCCCTCCACGAT	103
*RYBP*	TTGGCAGTGACTGTGGGCA	TGCTCTGGTTCTGCTGTTCTGAC	124

## Abbreviations

Rybp	RING1 and YY1 binding protein
AD-NC	Negative control adenoviruses
AD-Rybp	Rybp adenoviruses
DEDAF	Death effector domain-associated factor
PRC1	Polycomb repressive complex 1
LDM	Longissimus dorsi muscle
MOI	Multiplicity of infection
BH	Benjamini–Hochberg
BMTs	Body measurement traits
FDR	False discovery rate
DEGs	Differentially expressed genes
MRFs	Myogenic regulatory factors
GO	Gene ontology
qRT-PCR	Quantitative real-time PCR
MiRNA	MicroRNA
UTR	Untransliated region
NF-κB	Nuclear factor kappa-light-chain-enhancer of activated B cells
PPAR	Peroxisome proliferator-activated receptor
GAPDH	Glyceraldehyde 3-phosphate dehydrogenase
CDS	Coding sequence
